# Phonon‐Driven Tetrahedral Tilts Enable Ultralow Bulk Thermal Expansion and Interstitial Oxide‐Ion Migration in Phenacite Solid Electrolytes

**DOI:** 10.1002/advs.76792

**Published:** 2026-07-27

**Authors:** Xiaohui Li, Lu liang, Qilong Shi, Xiaoge Wang, Cheng Li, Sihao Deng, Lunhua He, Qiang Li, Kun Lin, Xianran Xing, Xiaojun Kuang

**Affiliations:** ^1^ Guangxi Key Laboratory of Electrochemical and Magnetochemical Functional Materials College of Chemistry and Bioengineering Guilin University of Technology Guilin P. R. China; ^2^ Beijing Advanced Innovation Center for Materials Genome Engineering Institute of Solid State Chemistry University of Science and Technology Beijing Beijing P. R. China; ^3^ College of Chemistry and Molecular Engineering Beijing National Laboratory for Molecular Science Peking University Beijing P. R. China; ^4^ Oak Ridge National Laboratory Neutron Sciences Directorate Oak Ridge Tennessee USA; ^5^ Songshan Lake Materials Laboratory Dongguan P. R. China; ^6^ Spallation Neutron Source Science Center Dongguan P. R. China; ^7^ Beijing National Laboratory for Condensed Matter Physics Institute of Physics Chinese Academic of Sciences Beijing P. R. China; ^8^ Future Energy Interdisciplinary Center Key Laboratory of Solid‐State Energy Conversion and Storage of Jiangxi Education Department School of Intelligent Manufacturing and Future Energy Gannan Normal University Ganzhou Jiangxi P. R. China

**Keywords:** interstitial oxide ion migration, local structure, negative thermal expansion, oxide‐ion electrolyte, tetrahedral tilt

## Abstract

Oxide‐ion solid electrolytes are enabling components for high‐temperature electrochemical technologies, including batteries, fuel cells, membrane reactors, sensors, and electrolyzers. The long‐standing challenge is the thermal‐expansion‐coefficients (TECs) mismatch between electrolytes and adjacent device components over broad operating temperature windows. Here, we demonstrate a design paradigm of phonon‐driven negative‐thermal‐expansion (NTE) framework can be leveraged not only to suppress thermal expansion but also to activate interstitial oxide‐ion migration through collective tetrahedral tilts. As a proof of concept, we report the first demonstration of interstitial oxide‐ion conduction in La‐doped flexible phenacite‐type NTE Zn_2_GeO_4_ featuring interconnected 4‐ and 6‐membered ring. The resulting material shows ultralow bulk TECs (α_V_ = 7.232 × 10^−6^ K^−1^, 298–1273 K) and near‐zero macroscopic linear thermal expansion (α_L_ = 0.307 × 10^−6^ K^−1^, 298–700 K), corresponding to the lowest reported bulk thermal‐expansion coefficient among oxide‐ion‐conducting solids. Interstitial‐oxygen species are accommodated within 4‐membered rings predominantly coordinated by GeO_4_ tetrahedra, and long‐range migration proceeds from 4‐ to 6‐membered rings, coupling the correlated interstitial‐oxygen disorder with the phonon‐driven NTE behavior involving collective tilts of the intrinsic flexible ZnO_4_ and GeO_4_ tetrahedra. This work pioneers the integration of NTE behavior with oxide‐ion transport, offering a promising strategy to mitigate TEC mismatch in solid‐state ionic devices.

## Graphical abstract

This work demonstrates a design paradigm of phonon‐driven negative‐thermal‐expansion (NTE) flexible framework that exploits the intrinsic lattice flexibility of NTE frameworks to simultaneously suppress thermal expansion and activate oxide‐ion conduction, offering a promising strategy to mitigate TEC mismatch in solid‐state ionic devices.

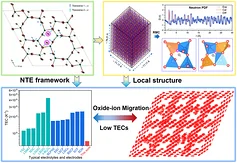

## 1 Introduction

Oxide‐ion solid electrolytes underpin a wide range of high‐temperature electrochemical and catalytic technologies, including fuel cells [1, 2, 3], membrane reactors [4, 5], high‐temperature sensors [6, 7], and electrolysers [8, 9]. A long‐standing fundamental challenge, however, is the mismatch in thermal‐expansion‐coefficients (TECs) between solid electrolytes and adjacent device components at broad operating temperatures, which leads to large internal strain gradients, delamination, and ultimately device failure [9, 10, 11]. The severity of this mismatch is well exemplified in solid oxide fuel cell (SOFC) architectures, where the widely used cobalt‐based perovskite cathodes exhibit large TECs of 20–25 × 10^−6^ K^−1^ [12, 13, 14], nearly double those of the typical electrolytes like samarium‐doped ceria SDC [15], and yttria‐stabilized zirconia YSZ [16] (11.2–12.3 × 10^−6^ K^−1^). Addressing this challenge therefore hinges on a critical materials challenge: how to design oxide‐ion‐conducting solids with intrinsically low‐ or even negative‐thermal expansion (LTE or NTE) over broad temperature windows.

Negative thermal expansion (NTE) materials provides a promising route for TEC engineering [17, 18, 19, 20]. Mechanistically, NTE behavior generally arises from two distinct origins: low‐frequency phonons associated with flexible framework dynamics, or electronic‐structure transitions [21, 22, 23, 24, 25]. Phonon‐driven NTE materials typically exhibit broad temperature windows of contraction due to cooperative lattice tilts and rigid‐unit modes, whereas NTE arising from electronic transitions often displays large contraction magnitudes but is confined to narrow temperature intervals. These characteristics highlight the particular promise of phonon‐driven flexible‐framework NTE systems as a design platform for achieving low or negative thermal expansion while simultaneously modulating oxygen‐defect formation and migration in solid electrolytes.

Interest in ionic conduction within NTE materials dates back to the 1990s, most notably in the A_2_(MO_4_)_3_ family (A = Sc^3+^, In^3+^, Al^3+^, or RE^3+^, M = W^6+^/Mo^6+^) [26, 27, 28, 29, 30, 31, 32, 33, 34]. However, these studies mainly concerned the identity of non‐oxide‐ion charge carriers, with the long‐standing controversy eventually resolved in favor of trivalent cation transport rather than oxide‐ion migration [35, 36, 37, 38, 39, 40]. Meanwhile, low‐temperature oxygen motion has been observed in NTE materials ZrW_2‐_ *_x_* Mo*_x_* O_8_ and attributed to the α‐to‐β phase transition, wherein the terminal W‐O species convert into corner‐sharing oxygen sites [41, 42]. However, such oxygen motion is confined within the short ranges, and no apparent long‐range oxide ion migration was realized in the NTE materials. These examples highlight that oxide‐ion‐conducting NTE solids remains in its infancy to date. In parallel, current strategies for mitigating TEC mismatch in fuel cells have focused either on reducing the TECs of device components or on introducing inert NTE phases into cell architectures [17, 43, 44, 45, 46, 47, 48], approaches that do not provide intrinsically active solid electrolytes combining low thermal expansion with oxide‐ion transport.

To meet the dual requirement for the low or negative thermal expansion and oxide‐ion conduction in solid electrolytes, conventional NTE phase alone is typically unable to meet both criteria, especially over a broad temperature window [49]. Motivated by the unique promise of phonon‐driven flexible NTE frameworks, we propose a new design paradigm that integrates oxide‐ion conducting functionality directly into such frameworks, enabling them to function as active oxide‐ion solid electrolytes, simultaneously with low‐ or negative TECs over a wide temperature range.

Open framework oxide‐ion solid electrolytes are natural candidates for this purpose because their structural openness can accommodate and transport oxygen defects [50, 51, 52]. Representative structural families include melilite [53], langasite [54, 55], perovskite‐derived [56, 57], apatite [58], mayenite [59], and more recently, zeolite‐like frameworks [60]. These open structural framework motifs, typically observed in the phonon‐driven flexible NTE frameworks, provide a guiding principle for engineering oxide ion‐conducting NTE materials.

Among known phonon‐driven NTE oxides, phenacite‐type Zn_2_(Ge/Si)O_4_ is particularly attractive because it contains interconnected 4‐ and 6‐membered rings built from tetrahedral units [61, 62]. Both the Zn_2_GeO_4_ [61] and Zn_2_SiO_4_ [62] adopt an AB_2_O_4_ framework composed of ZnO_4_ and SiO_4_/GeO_4_ tetrahedra within the (001) plane, along with 3‐membered rings oriented perpendicular to it. In contrast to the rigid Zn_2_SiO_4_ structural framework, Zn_2_GeO_4_ offers greater structural flexibility owing to the variable coordination of Ge^4+^ (CN = 4–6).

Herein, we report the first demonstration of interstitial oxide ion conduction into the flexible NTE framework of Zn_2_GeO_4_ via substitution of Zn^2+^ with La^3+^. The accommodation and transport mechanisms of interstitial oxygen, and its coupling with NTE behavior in the NTE structural framework of Zn_2‐_ *_x_* La*_x_* GeO_4+_ *_x_*_/2_ were systematically elucidated through fine average and local structural analysis, as well as molecular dynamics (MD) simulations. The interstitial oxide ion conducting NTE materials Zn_2‐_ *_x_* La*_x_* GeO_4+_ *_x_*_/2_ exhibit ultralow bulk TEC values (α_V_ = 7.232 × 10^−6^ K^−1^, α_a_ = α_b_ = 1.823 × 10^−6^ K^−1^, α_c_ = 3.570 × 10^−6^ K^−1^, 298–1273 K) and near‐zero macroscopic linear expansion (α_L_ = 0.307 × 10^−6^ K^−1^, 298–700 K) over a broad‐temperature window. This study highlights a phonon–tilt–migration coupling strategy for developing LTE or NTE oxide‐ion solid electrolytes that simultaneously offer TEC engineering and oxide‐ion conduction, thereby opening a new avenue for addressing long‐standing thermo‐mechanically incompatibilities in solid‐state ionic devices.

## 2 Experimental Section

### 2.1 Material Synthesis

Polycrystalline powders of Zn_2‐_ *_x_* La*_x_* GeO_4+_ *_x_*_/2_ (0 ≤ *x* ≤ 0.1) were synthesized by a conventional high‐temperature solid‐state reaction method. La_2_O_3_ (99.99%, Aladdin), ZnO (99.99%, Aladdin), and GeO_2_ (99.99%, Aladdin) were used as the starting materials. The La_2_O_3_ powder was pre‐fired at 950°C for 2 h before being weighed to remove the absorbed H_2_O and CO_2_. The stoichiometric amounts of La_2_O_3_, ZnO, and GeO_2_ were weighed and mixed in a pestle and mortar using ethanol as the mixing media to obtain uniform mixtures. The mixed powders were pre‐sintered at 1000°C for 10 h, then reground well, pressed into pellets, and transferred to alumina crucibles and fired at 1300°C for 10 h to obtain the final ceramics with the relative densities of ∼ 87%.

### 2.2 Material Characterization

The phase formation of Zn_2‐_ *_x_* La*_x_* GeO_4+_ *_x_*_/2_ (0 ≤ *x* ≤ 0.1) was checked by powder X‐ray diffraction (PXRD) using a Rigaku SmartLab 9 kW diffractometer with Cu Kα_1_ radiation, operating at 40 kV and 135 mA. Variable temperature XRD data in a temperature range of 25°C–1000°C were collected on a PANalytical X'Pert PRO X‐ray diffractometer equipped with an Anton Parr HTK 1200N high‐temperature attachment. Time‐of‐flight (TOF) neutron powder diffraction (NPD) data were collected on a general‐purpose powder diffractometer (GPPD) at the China Spallation Neutron Source (CSNS) [63], TOPAS Academic software [64] and JANA 2006 software [65] were used to perform Rietveld analysis of the XRD and NPD data. The rotation electron diffraction (RED) data was collected on a JEOL‐2100 electron microscope operating at 200 kV using a single‐tilt tomography sample holder, and REDp [66], processing software was used to process the RED data. The total scattering neutron pair distribution function (nPDF) data were performed on the BL‐1B NOMAD beamlines at Oak Ridge National Laboratory of the Spallation Neutron Source (SNS). The total scattering X‐ray pair distribution function (XPDF) data were collected at the BL13SSW beamline at the Shanghai Synchrotron Radiation Facility (SSRF). The average lattice fitting was conducted based on PDFgui software [67]. Reverse Monte Carlo simulations for the XPDF and nPDF data were carried out by RMCprofile software [68], combining the atomic movements and swapping, which utilizes a 5 × 5 × 8 (71.17 Å × 71.17 Å × 76.21 Å) large supercell containing 25400 atoms.

Morphologies and energy dispersive X‐ray spectroscopy data were collected using a FEI Tecnai‐G2 field emission transmission electron microscope (TEM) equipped with a Li/Si EDX detector. Thermogravimetric analysis and differential scanning calorimetry (TG‐DSC) data were recorded on a high‐temperature platform 400 (STA 449 F3 Jupiter, NETZSCH‐Gerätebau GmbH) simultaneous analyzer. Alternating‐current (AC) impedance spectroscopy measurements were carried out on a Solartron 1260 frequency response analyzer over a frequency range of 10^−1^–10^7^ Hz. Pt paste was used as electrodes, and the experimental complex impedance data were analyzed by ZView software [69]. The oxygen transport numbers ($t_{o^{2-}}$) were obtained by monitoring the electromotive force (EMF) of measured ceramic using the oxygen concentration method in a temperature range of 500°C–1000°C. The measured pellet was sealed at the tip of an alumina tube, and two surfaces of the pellet were exposed to pure O_2_ flowing and 1% O_2_ flowing, respectively. Linear thermal expansion measurements were collected on an advanced thermal dilatometer (NETZSCHDIL402) in the temperature range from 298 to 700 K with a heating rate of 5 °C/min.

#### 2.2.1 Bond‐Valence Site Energy (BVSE) Calculations

BVSE calculations based on the principle of local charge neutrality were carried out on the SoftBV program [70], and the calculated oxide ions were placed at all points of the whole cell. The oxide ion migration pathways were obtained by calculating bond‐valence‐based energy landscapes, which show the pathways connecting local minima and saddle points, i.e., the migration pathways were determined based on the regions of low BVSE connecting local minima and saddle points.

Phonon calculations were performed using first‐principles density functional theory as implemented in VASP [71]. The PAW method was used to describe the ion‐electron interactions, and the exchange–correlation energy was treated using the PBE functional [72]. A plane‐wave energy cutoff of 520 eV and a 3 × 3 × 5 Monkhorst‐Pack *k*‐point mesh were used to sample the Brillouin zone. Prior to the phonon calculations, the structure was fully relaxed until the electronic energy and residual forces converged to 10^−8^ eV and 0.001 eV Å^−1^, respectively. The phonon dispersion and projected phonon density of states were calculated using density functional perturbation theory (DFPT) to obtain the dynamical matrix [73]. The phonon dispersion and projected phonon density of states were then generated using Phonopy [74].

#### 2.2.2 Molecular Dynamics (MD) Simulations

Atomistic static lattice simulations of Zn_2‐_ *_x_* La*_x_* GeO_4_ were carried out using the General Utility Lattice Program (GULP) on the basis of the interatomic potential approach [75]. The Buckingham potential function [76], was used to model ion interactions, incorporating the shell model to account for electronic polarizability. The potential parameters of La‐O, Zn‐O, Ge‐O, and O‐O used for the atomistic simulation are listed in Table 1 [77, 78]. The MD simulations were performed using the DL_POLY code in the NVT ensemble [79]. The initial simulation cell was constructed from a 4 × 4 × 4 supercell of the phenacite‐type Zn_2_GeO_4_, containing 2696 atoms (including the shell model), including 752 Zn atoms, 384 Ge atoms, 16 La atoms and 1544 O atoms. La dopants were introduced by substituting Zn sites, and charge compensation was achieved by adding oxygen interstitials according to the defect chemistry of La‐doped Zn_2_GeO_4_. The initial interstitial oxygen sites were selected based on the interstitial positions determined from NPD analysis. In the starting configuration, the La dopants and oxygen interstitials were randomly distributed within the simulation box. The resulting model, Zn_752_La_16_Ge_384_O_1544_, corresponds to the nominal composition of Zn_1.96_La_0.04_GeO_4.02_. Before the production MD runs, the structures were first energy‐minimized through zero‐temperature dynamic simulations for 100 ps, and then equilibrated under a constant pressure of 1 atm for 20 ps. The MD simulations were subsequently performed within the temperature range of 273–1473 K. Each simulation was run for 2500000 steps with a time step of 0.04 fs, corresponding to a total simulation time of 100 ps. MD data analysis was conducted using Visual Molecular Dynamics (VMD) packages [80], complemented by the nMoldyn code [81], to extract the mean square displacement (MSD) data. Oxygen diffusion coefficients were determined by linear fitting to the MSD vs. time plots.

**TABLE 1:** Buckingham interatomic potential and shell model parameters for the Zn_2_GeO_4_‐based materials.

Interaction	*A* (eV)	*ρ* (Å)	*C* (eV Å^6^)	*Y*(e)	*K* (eV Å^−2^)
Zn‐O	529.7	0.3581	0	2	10.28
Ge‐O	1479.9667	0.325647	16.808599	4	317.2
La‐O	4579.23	0.30437	0	3	99999
O‐O	22764.3	0.149	27.89	−2.869	74.92

## 3 Results and Discussion

### 3.1 Solid‐Solution Formation of Zn_2‐_ *_x_* La*_x_* GeO_4+_ *_x_*_/2_ Electrolytes

To introduce interstitial oxygen defects into the flexible NTE framework, we aliovalently substituted La^3+^ for Zn^2+^ in phenacite‐type Zn_2_GeO_4_. Figure 1a shows the XRD patterns of Zn_2‐_ *_x_* La*_x_* GeO_4+_ *_x_*_/2_ (0 ≤ *x* ≤ 0.1), revealing that when *x* ≥ 0.06, weak reflections attributable to the La_2_Ge_2_O_7_ impurity phase appeared. To accurately identify the solid solution limit, Rietveld analysis was carefully performed for both Zn_1.96_La_0.04_GeO_4.02_ (Figure 1b, x = 0.04) and Zn_1.95_La_0.05_GeO_4.025_ (Figure S1, *x* = 0.05) compositions, which revealed that the *x* = 0.05 composition contained minor impurity peaks (*R*_wp_ = 9.91%, Figure S1), and the *x* = 0.04 composition shows a well fit (*R*_wp_ = 8.817%, Figure 1b and Table S1) without apparent impurity reflections. This suggests that *x*  =  0.04 is the solubility limit for Zn_2‐_ *_x_* La*_x_* GeO_4+_ *_x_*_/2_. The refined lattice parameters (Figure 1c) increase linearly with *x* up to 0.04, further supporting *x*  =  0.04 as the upper limit of homogeneity. Three‑dimensional continuous rotation electron diffraction (3D cRED, Figure 1d–g) confirmed that Zn_2‐_ *_x_* La*_x_* GeO_4+_ *_x_*_/2_ adopt a trigonal unit cell (Space group: *R* $\bar{3}$, *a* = *b* = 13.06 Å, *c* = 9.46 Å, *α* = *β* = 90°, *γ* = 120°) with extinction conditions of (*0kl*): *l* = 3n, and (*h0l*): *h* = 3n. TEM elemental mapping (Figure 1h, Figures S2 and S3) of Zn_1.96_La_0.04_GeO_4.02_ shows a uniform distribution for Zn, La, Ge, and O throughout the crystals. These results collectively indicate successful incorporation of La^3+^ into the NTE Zn_1.96_La_0.04_GeO_4.02_ lattice and the concomitant formation of interstitial oxygen defects, without inducing phase segregation.

**FIGURE 1:**
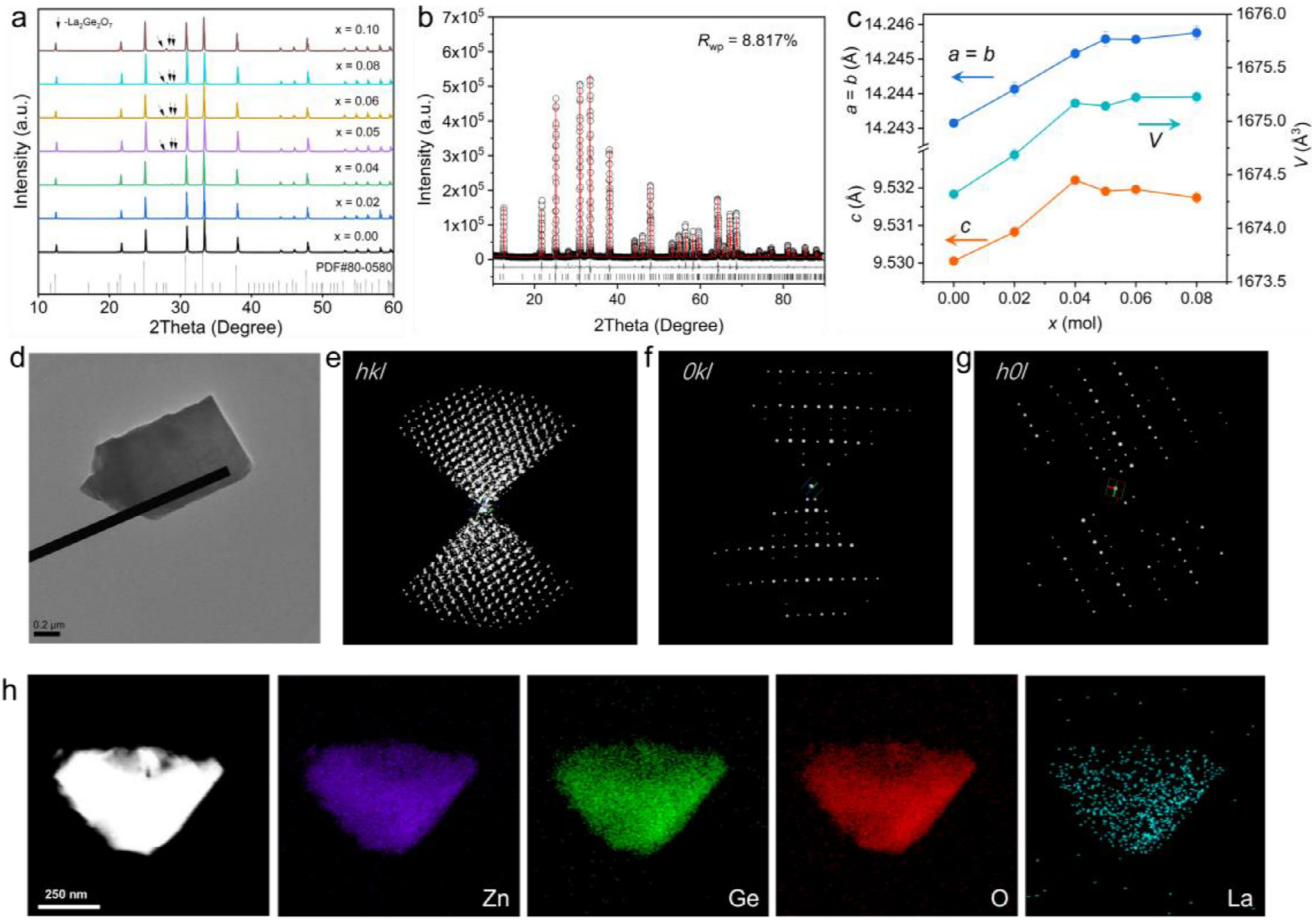
Solid solution formation of Zn_2‐_ *_x_* La*_x_* GeO_4+_ *_x_*_/2_. (a) XRD patterns for compositions 0 ≤ *x* ≤ 0.1. (b) Rietveld refinement plots of XRD pattern for Zn_1.96_La_0.04_GeO_4.02_. (c) Cell parameters as a function of *x*. (d) TEM image and (e–g) 3D cRED data: (e) reconstructed reciprocal lattice, (f) (*0kl*), and (g) (*h0l*) diffraction planes. (h) TEM‐elemental mapping of Zn_1.96_La_0.04_GeO_4.02_.

### 3.2 Electrical and Thermal Expansion Properties

To study the effect of La^3+^ doping on the electrical properties of Zn_2‐_ *_x_* La*_x_* GeO_4+_ *_x_*_/2_, alternating current (AC) impedance spectroscopy measurements were performed. Unlike the parent Zn_2_GeO_4_ (Figure S4), which displays only bulk and grain boundary responses, the typical complex impedance plots of Zn_1.96_La_0.04_GeO_4.02_ at 500°C and 600°C (Figure 2a, Figure S5) exhibit remarkable Warburg responses, indicative of significant ionic diffusion. Notably, the inclined Warburg‐type electrode response arc for Zn_1.96_La_0.04_GeO_4.02_ observed at low temperatures (Figure S5) gradually collapses down to a semicircular arc at elevated temperatures (Figure 2b), characteristic of typical oxide ion conduction (Supplementary note 1), which could be owing to the creation of interstitial oxide ion defects, as described by the following defect reaction:


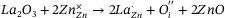




**FIGURE 2:**
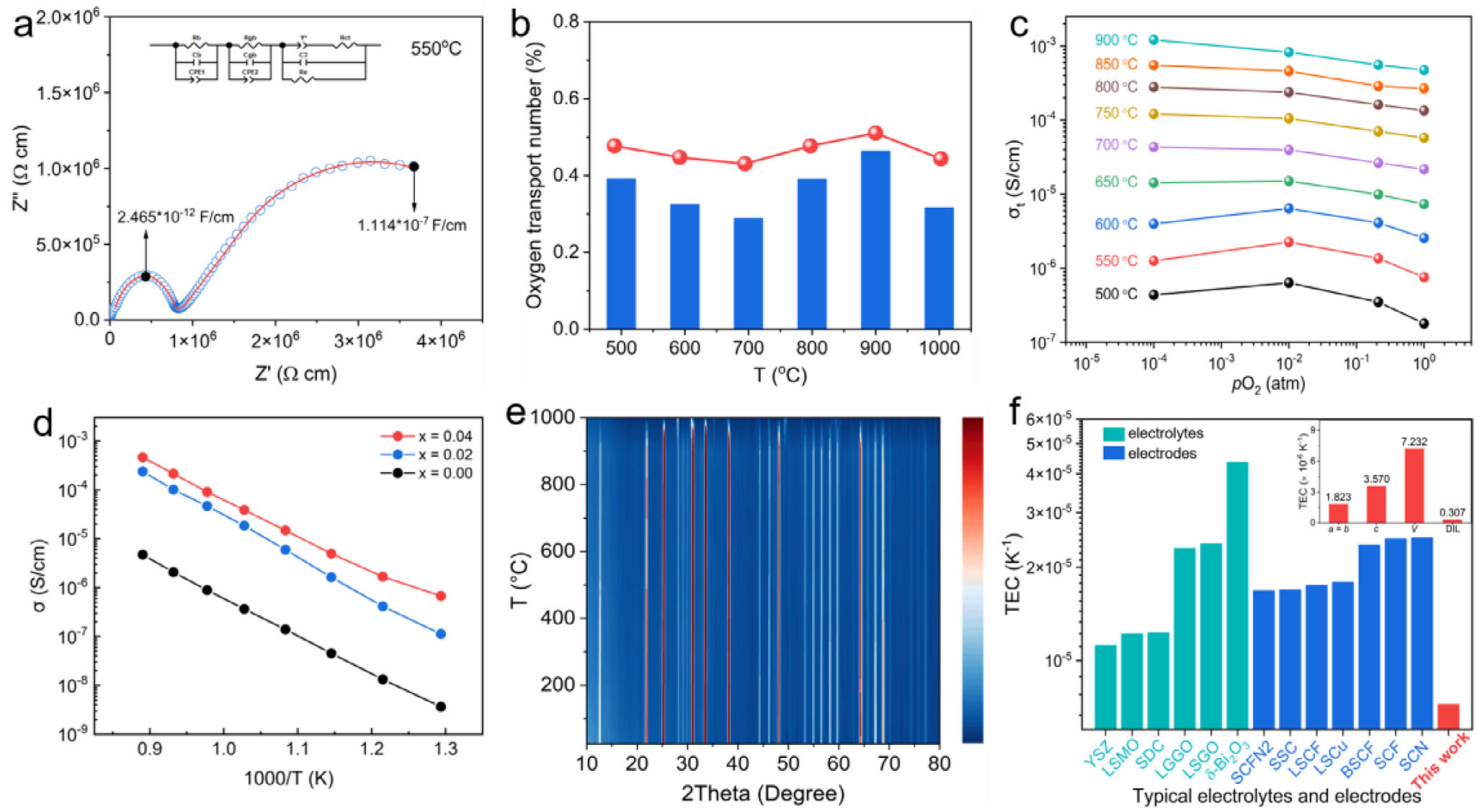
Electrical and thermal expansion properties of Zn_2‐_ *_x_* La*_x_* GeO_4+_ *_x_*_/2_. (a) Complex impedance plots of Zn_1.96_La_0.04_GeO_4.02_ at 550°C with the equivalent circuits used for fitting. (b) Oxygen transport numbers of Zn_1.96_La_0.04_GeO_4.02_ ceramic. (c) Conductivities of Zn_1.96_La_0.04_GeO_4.02_ as a function of oxygen partial pressures. (d) Bulk conductivities of Zn_2‐_ *_x_* La*_x_* GeO_4+_ *_x_*_/2_ (0 ≤ *x* ≤ 0.04) in air. (e) Variable‐temperature XRD patterns of Zn_1.96_La_0.04_GeO_4.02_ in the temperature range of 25°C–1000°C. (f) TECs of Zn_1.96_La_0.04_GeO_4.02_ in comparison with the representative SOFC electrolytes and electrodes. Electrolytes: YSZ (8 mol% Y_2_O_3_‐stabilized ZrO_2_), LSMO (La_0.8_Sr_0.2_Ga_0.8_Mg_0.2_O_3‐δ_), SDC (Sm‐doped CeO_2_), LGGO (La_3_Ga_5+_ *_x_* Ge_1‐_ *_x_* O_14‐_ *_x_*_/2_), and melilite LSGO (La_1.54_Sr_0.46_Ga_3_O_7.27_); Electrodes: SCFN2 (Sr_0.9_Ce_0.1_Fe_0.8_Ni_0.2_O_3‐δ_), SSC (SrSc_0.2_Co_0.8_O_3‐δ_), LSCF (La_0.6_Sr_0.4_Co_0.2_Fe_0.8_O_3‐δ_), LSCu (La_0.7_Sr_0.3_CuO_3‐δ_), BSCF (Ba_0.5_Sr_0.5_Co_0.8_Fe_0.2_O_3‐δ_), SCF(SrCo_0.7_Fe_0.2_W_0.1_O_3‐δ_), and SCN (SrCo_1‐_ *_x_* Nb*_x_* O_3‐δ_). Inset: TECs of Zn_1.96_La_0.04_GeO_4.02_ determined by VT‐XRD and dilatometry.

The depressed semicircular arcs observed in the complex impedance data (Figure 2a, Figures S4 and S5) indicate non‐ideal electrochemical behavior of parallel resistive (*R*) and capacitive (*C*) elements, arising from the frequency‑dependent variations in the R and C elements and overlapping of multiple relaxation processes [82]. Therefore, the data were fitted using an equivalent circuit comprising a resistor, capacitor, and constant‑phase element (*CPE*) (*R*//*C*//*CPE*; insets of Figure 2a). The inclusion of the *CPE* admittance as a frequency‐dependent term accounts for high‐frequency dispersion in conductivity and low‐frequency dispersion in capacitance [83, 84, 85].

The complex impedance plots of Zn_1.96_La_0.04_GeO_4.02_ under different atmospheres (pure O_2_, Air, 1%O_2_/99%Ar, Figure S6) show that the resistance decreases with decreasing oxygen partial pressures (*p*O_2_, 1–10^−2^ atm), indicative of the presence of *n*‐type electronic conduction at 600°C under the high *p*O_2_ range. The oxygen transport number of Zn_1.96_La_0.04_GeO_4.02_ ceramic was determined via the oxygen concentration cell method (Figure 2b), which is approximately 35% across the 500°C–1000°C temperature range, demonstrating the mixed electronic and oxide ionic conduction in Zn_1.96_La_0.04_GeO_4.02_. In addition, *p*O_2_‐dependent conductivities of Zn_1.96_La_0.04_GeO_4.02_ (Figure 2c) reveal that the material exhibits dominant *n*‐type conduction behavior. Bulk conductivities of Zn_2‐_ *_x_* La*_x_* GeO_4+_ *_x_*_/2_ (0 ≤ *x* ≤ 0.1, Figure 2d) show an enhancement of nearly two orders of magnitude with increasing interstitial oxygen concentration, accompanied by a decrease in activation energy from 1.61 eV for the parent composition (*x* = 0) to 1.29 eV for *x* = 0.04 composition, highlighting the critical role of the introduced interstitial oxygen in promoting the oxide‐ion transport.

To evaluate the thermal expansion behavior of the oxide ion‐conducting material Zn_1.96_La_0.04_GeO_4.02_, variable temperature XRD (VT‐XRD) combined with linear thermal expansion measurements were performed for Zn_1.96_La_0.04_GeO_4.02_. Figure 2e (see more details in Figure S7) shows the VT‐XRD patterns of Zn_1.96_La_0.04_GeO_4.02_ in the temperature range of 25°C–1000°C, indicating the thermal stability of this material over the broad temperature windows, as also evidenced by TG‐DSC data (Figure S8). The refined lattice parameters (Figure S9) yield ultralow bulk TECs in the broad temperature window of 298–1273 K, with the bulk TEC values (Inset of Figure 2f) of α_V_ = 7.232 × 10^−6^ K^−1^, α_a_ = α_b_ = 1.823 × 10^−6^ K^−1^, α_c_ = 3.570 × 10^−6^ K^−1^. Complementary linear thermal expansion measurement by a dilatometer (Inset of Figure 2f, Figure S10) gives a near‐zero macroscopic linear thermal expansion over a broad temperature window (α_L_ = 0.307 × 10^−6^ K^−1^, 298–700 K). Notably, to the best of our knowledge, this α_V_ value represents the lowest reported bulk thermal‐expansion coefficient among oxide‐ion‐conducting solids.

In contrast to commonly used electrolytes and electrodes having much higher TECs (electrolytes:11.2–43.6 × 10^−6^ K^−1^; electrodes: 16.8–25.0 × 10^−6^ K^−1^, Figure 2f), such as SrSc_0.2_Co_0.8_O_3‐δ_ (SSC) [86], (La, Sr)(Co, Fe)O_3‐δ_ (LSCF) [87, 88], Ba_0.5_Sr_0.5_Co_0.8_Fe_0.2_O_3–δ_ (BSCF) [89], and SrCo_0.9_Nb_0.1_O_3‐δ_ (SCN) [90, 91], here the oxide‐ion conducting negative‐thermal‐expansion (NTE) material Zn_1.96_La_0.04_GeO_4.02_ displays exceptionally low TECs. More critically, whereas the approach of integrating a single inert NTE phase with device components solely mitigates thermal mismatch, our strategy advances a new promising strategy for developing LTE or NTE oxide‐ion solid electrolytes that simultaneously offer TEC engineering and oxide‐ion conduction.

### 3.3 Coupling Interstitial Oxide‐Ion Migration with NTE Behavior

#### 3.3.1 Location of Interstitial Oxygen in the Average Structure

Although the incorporation of interstitial oxygen into the Zn_2‐_ *_x_* La*_x_* GeO_4+_ *_x_*_/2_ framework enhances the oxide ionic conductivity, the overall ionic‐transport performance remains modest. To elucidate the underlying structure‐property relationship in Zn_2‐_ *_x_* La*_x_* GeO_4+_ *_x_*_/2,_ we collected room‐temperature neutron powder diffraction (NPD) data on Zn_1.96_La_0.04_GeO_4.02_ to determine the interstitial oxygen position and analyze the local chemical bonding environment. Rietveld analysis of NPD data of Zn_1.96_La_0.04_GeO_4.02_ using the trigonal structural model (Space group: *R* $\bar{3}$) shows a well fit (*R*_wp_ = 4.00%, *R*_p_ = 3.23%, and GOF = 3.72). During the Rietveld refinement, the La occupancies were allowed on both crystallographically distinct Zn sites (Zn1 and Zn2), revealing the preferential La^3+^ occupancy on the Zn1 site (*occ* = 0.05(2), Table 2). This preferential La^3+^ occupancy is also consistent with the Rietveld analysis result of XRD data (*occ* = 0.06(3), Table S1).

**TABLE 2:** The final refined structural parameters of Zn_1.96_La_0.04_GeO_4.02_ from NPD data^a^.

Atom	Wyckoff site	*x*	*y*	*z*	Occupancy	U_iso_(Å^2^)
Zn1	18*f*	−0.217(2)	−0.025(2)	−0.590(2)	0.95 (2)	0.007(2)
La1	18*f*	−0.217(2)	−0.025(2)	−0.590(2)	0.05 (2)	0.007(2)
Zn2	18*f*	−0.2052(18)	−0.019(2)	−0.925(2)	1	0.008(5)
Ge1	18*f*	−0.2176(15)	−0.0207(15)	−0.252(2)	1	0.005(2)
O1	18*f*	−0.210(2)	−0.089(2)	−0.087(3)	1	0.007(4)
O2	18*f*	−0.2190(15)	−0.0908(18)	−0.396(2)	1	0.007(2)
O3	18*f*	−0.2081(19)	−0.0960(18)	−0.748(3)	1	0.004(1)
O4	18*f*	−0.327(2)	−0.001(3)	−0.263(2)	1	0.008(3)
O_i_	18*f*	−0.22(4)	0.09(3)	−1.33(3)	0.036(6)	0.03(2)

^a^ Space group: *R* $\bar{3}$, *a* = *b* = 14.2033(6) Å, *c* = 9.5064(6) Å, *α* = *β* = 90°, *γ* = 120°, *V* = 1660.8(1) Å^3^.

Through combined Rietveld analysis and charge flipping method, we identified a weak residual nuclear scattering density corresponding to interstitial oxygen O_i_ at the 18*f* Wyckoff site (coordinates: −0.22(4), 0.09(3), −1.33(3), marked by cyan in Figure 3b). Incorporation of this O_i_ site into the Rietveld refinement improved the fit (Figure 3a and Table 2) with *R*‐factors of *R*_wp_ = 3.78%, *R*_p_ = 2.47%, and GOF = 3.48. The refined structure (Figure 3c) shows that the interstitial oxygen was accommodated within the 4‐membered rings, coordinated by Ge1O_4_ and Zn1O_4_ tetrahedra, leading to (Zn1, Ge1)O_8_ edge‐sharing polyhedral dimer unit (Figure 3d), rather than by Zn2O_4_ tetrahedra (Figure 3e). Consistent with the preferential La^3+^ substitution at the Zn1 site, the interstitial oxygen O_i_ is located closer to the Zn1O_4_ environment rather than to Zn2O_4_. The refined Zn1‐O_i_ and Ge1‐O_i_ bond lengths are 1.9(4) Å and 1.7(4) Å, respectively, with the shorter Ge1‐O_i_ bond reflecting the more covalent character of Ge─O bond than the Zn─O bond. The relatively large uncertainties in the bond lengths arise from the weak neutron scattering signal associated with the low concentration of interstitial oxygen. The stronger bonding of O_i_ with Ge could confine the mobility of interstitial oxide ions, which is further understood based on the local structure analyzed by using X‐ray and neutron pair distribution function techniques.

**FIGURE 3:**
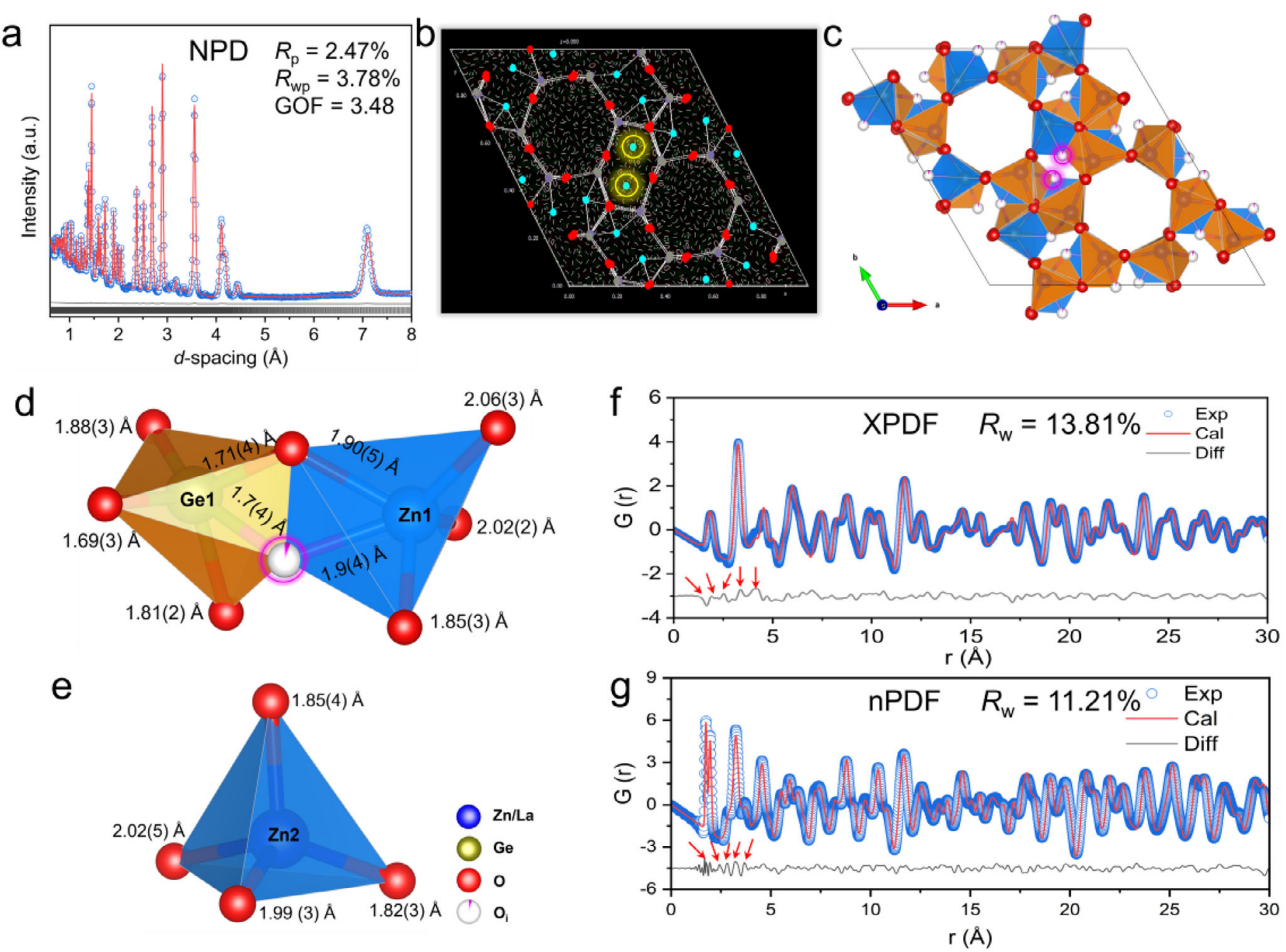
Average structure of Zn_1.96_La_0.04_GeO_4.02_. (a) Rietveld refinement plots of NPD data. (b) Fourier difference nuclear density map viewed along [001] direction, where the cyan sphere denotes the interstitial oxygen O_i_. (c) The refined average structure containing interstitial oxygen O_i_ highlighted by red circles. (d, e) Enlarged coordination environments around (d) Ge1 and Zn1, and (e) Zn2 cations. (f, g) Small box refinements for the (f) XPDF and (g) nPDF data.

To unveil the local structural variations in Zn_2‐_ *_x_* La*_x_* GeO_4+_ *_x_*_/2_ induced by interstitial oxygen incorporation, X‐ray and neutron total scattering based on the pair distribution function (XPDF and nPDF) techniques were employed. Small‐box refinement of the XPDF (Figure 3f) and nPDF (Figure 3g) data for Zn_1.96_La_0.04_GeO_4.02_ based on the average structural model reveals distinct mismatches within the 0–5 Å real‐space range, as marked by the red arrows (Figure 3f,g), indicating short‑range local structural disorder or distortions. These structural deviations, not captured in the average structure from small‐box refinement, are closely associated with oxide‑ion mobility and the intrinsic NTE behavior.

#### 3.3.2 Correlated Disorder of Interstitial Oxygen in the Local Structure

To capture the local variation of atomic distributions within Zn_1.96_La_0.04_GeO_4.02_, we performed reverse Monte Carlo (RMC) simulations against the XPDF (Figure 4b) and nPDF (Figure 4c) data, employing a large 71.17 Å × 71.17 Å × 76.21 Å supercell (Figure 4a). The RMC‐derived coordination‐number distributions (Figure S11) show average oxygen coordination numbers of 3.98, 4.02, and 3.99 for Zn, La, and Ge, respectively, confirming that the refined big‐box model preserves chemically reasonable local cation‐oxygen environments. By tracing the interstitial oxygens and their surrounding coordination polyhedra, the RMC‐derived structure demonstrates three discrete and locally preferred environments for interstitial oxygens (Figure 4d–f): (i) coordination with one GeO_4_ and one ZnO_4_ tetrahedron, forming a distorted edge‐sharing (Zn, Ge)O_8_ unit (Figure 4d), consistent with the coordination environment identified in the average NPD structure; (ii) coordination with two GeO_4_ and one ZnO_4_ tetrahedra (Figure 4e), embedding it within a predominantly GeO_4_ environment; and (iii) coordination with two GeO_4_ and two ZnO_4_ tetrahedra (Figure 4f). These findings highlight that the correlated disorder of interstitial oxygen with the GeO_4_ environment, together with the cooperative tilts of the surrounding GeO_4_ and ZnO_4_ tetrahedra, govern and trap the oxide‑ion mobility within the NTE Zn_2‐_ *_x_* La*_x_* GeO_4+_ *_x_*_/2_ framework.

**FIGURE 4:**
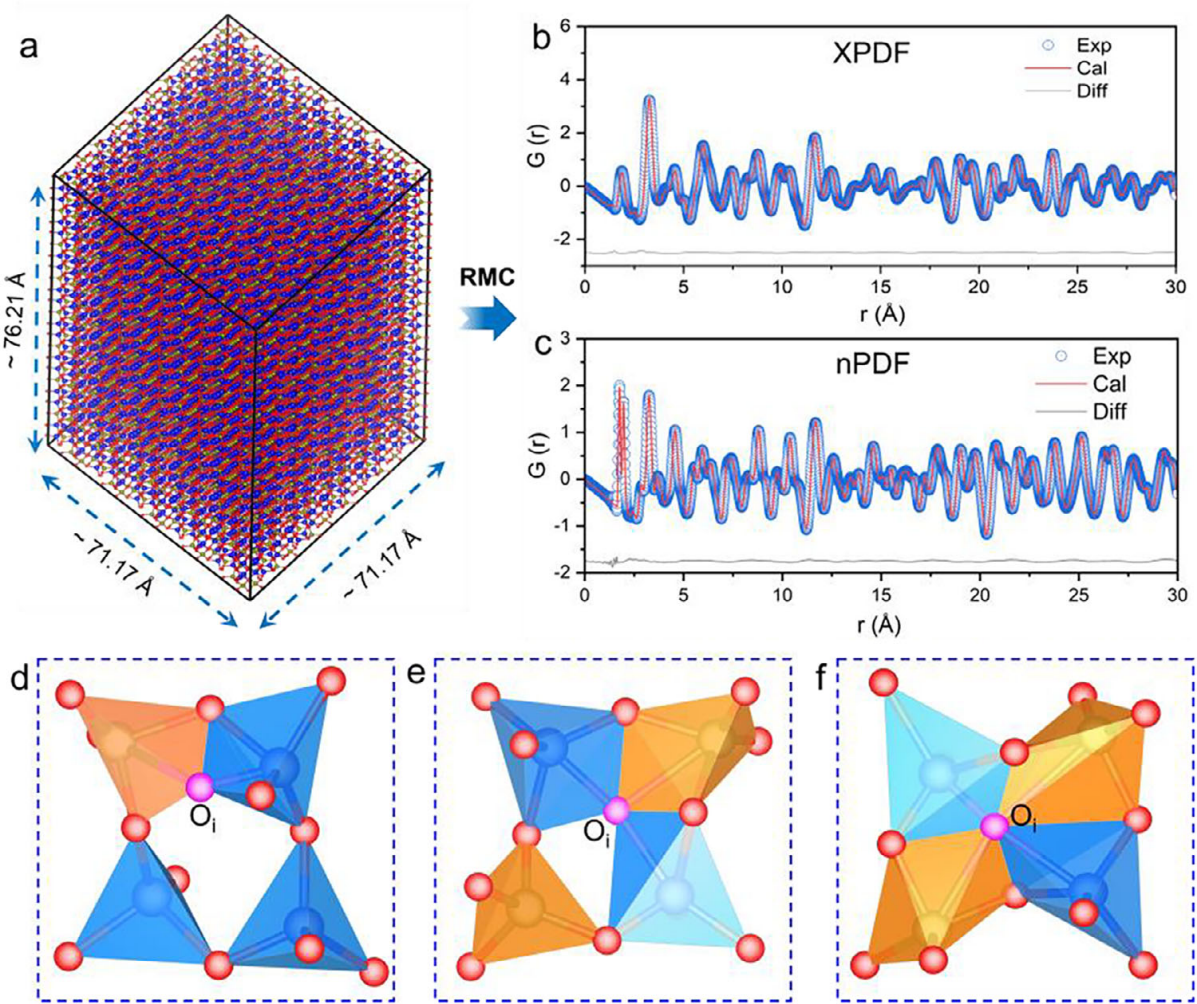
Local structure of Zn_2‐_ *_x_* La*_x_* GeO_4+_ *_x_*_/2_. (a) Three‐dimensional atomic configuration within an approximately 71.17 Å × 71.17 Å × 76.21 Å supercell used for RMC simulation. (b, c) Big‐box refinements for the (b) XPDF and (c) nPDF data. (d–f) Coordination environments of interstitial oxygen derived from RMC modeling: coordinated with (d) one Zn and one Ge, (e) two Zn and one Ge, and (f) two Zn and two Ge.

#### 3.3.3 NTE Behavior and Interstitial Oxygen Defect Coupling

Understanding the coupling between negative thermal expansion (NTE) behavior and interstitial oxide‐ion migration is crucial for the rational design of multifunctional NTE oxide‐ion solid electrolytes. In the La‐doped Zn_2‐_ *_x_* La*_x_* GeO_4+_ *_x/_*_2_ framework, anisotropic displacement parameter (ADP) analysis (Figure 5a) reveals pronounced transverse thermal vibrations of oxygen atoms within the *ab* plane and perpendicular to the *c*‐axis. Such anisotropic, temperature‐activated displacements promote cooperative tilting of the ZnO_4_ and GeO_4_ tetrahedra, resulting in the exceptionally low positive TEC values (α_V_ = 7.232 × 10^−6^ K^−1^, α_a_ = α_b_ = 1.823 × 10^−6^ K^−1^, α_c_ = 3.570 × 10^−6^ K^−1^, 298–1273 K), consistent with the vibrational behavior of the parent Zn_2_GeO_4_ framework [61].

**FIGURE 5:**
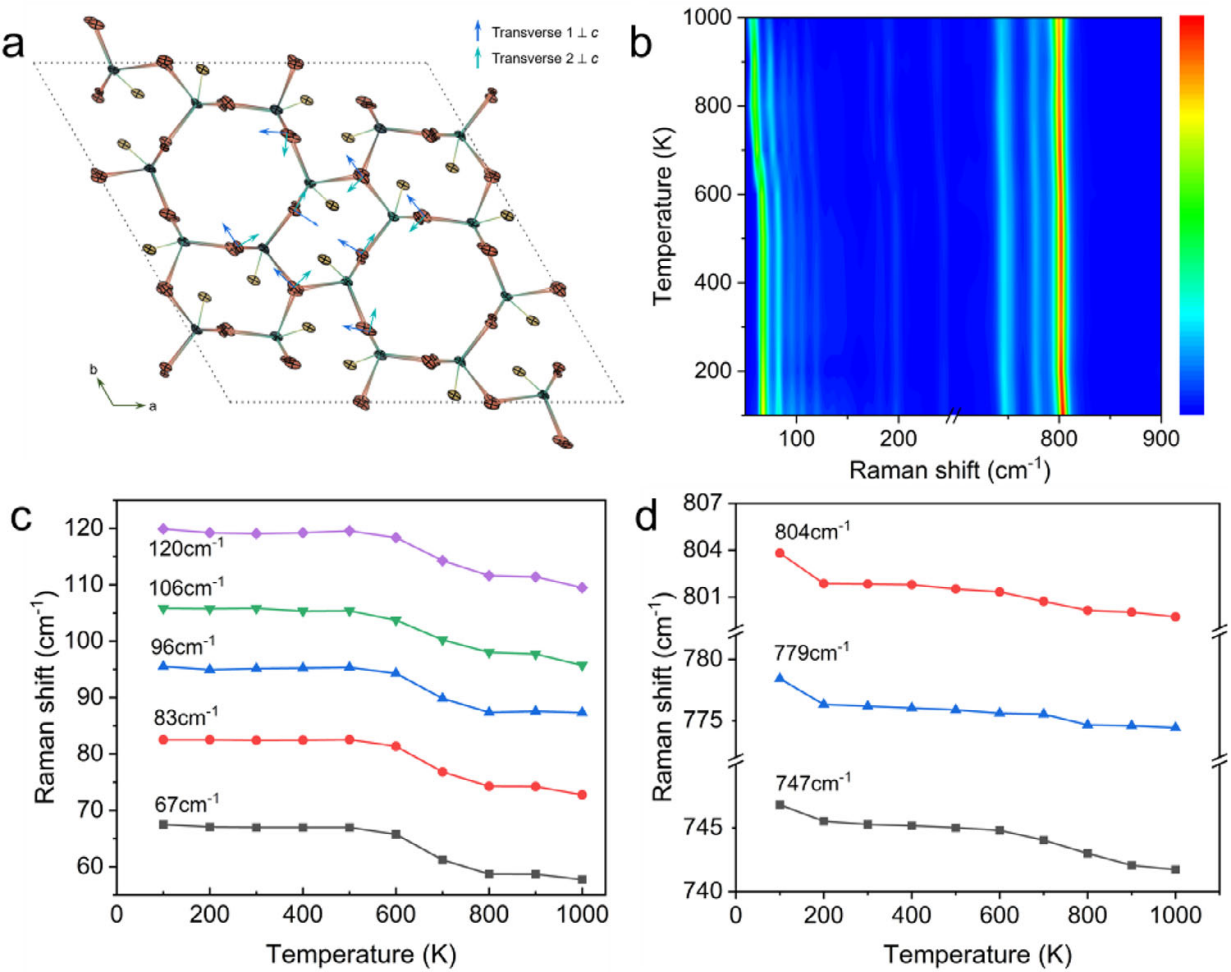
Coupling between NTE and interstitial oxygen defect in Zn_2‐_ *_x_* La*_x_* GeO_4+_ *_x_*_/2_. (a) Atomic displacement ellipsoid plots of the Zn_2‐_ *_x_* La*_x_* GeO_4+_ *_x_*_/2_ framework atoms refined from NPD data. (b) Temperature‐dependent Raman spectra collected between 100 and 1000 K. (c) Low‐ and (d) high‐wavenumber Raman modes as a function of temperature.

Variable‐temperature Raman spectroscopy (Figure 5b) provides complementary evidence for the phonon modes underlying the NTE behavior in Zn_2‐_ *_x_* La*_x_* GeO_4+_ *_x/_*_2_ framework. The low‐wavenumber modes (Figure 5c) primarily correspond to external vibrations of the GeO_4_ and ZnO_4_ tetrahedra, whereas the bands at ∼747, 779, and 805 cm^−1^ (Figure 5d) originate from O‐Ge‐O stretching, Ge‐O‐Zn bending, and O‐Ge‐O stretching, respectively [92, 93, 94]. Upon heating, high‐frequency Raman bands (Figure 5b,d) exhibit a systematic redshift, while low‐frequency modes (Figure 5b,c) initially blue‐shift and subsequently red‐shift. This anomalous evolution, characteristic of Zn_2‐_ *_x_* La*_x_* GeO_4+_ *_x/_*_2_‐type NTE frameworks, indicates that NTE behavior is governed by low‐frequency transverse modes involving collective motions of the tetrahedral units [93, 95]. Additional phonon calculations show no imaginary phonon branches in the calculated dispersion (Figure S12a), indicating the dynamic stability of the Zn_2_GeO_4_ framework. The projected phonon density of states (Figure S12b) reveals substantial Zn and O contributions in the low‐frequency region, indicating that these modes are closely associated with transverse oxygen motions and collective ZnO_4_/GeO_4_ tetrahedral tilting. This result is consistent with previous phonon and Grüneisen‐parameter analyses of Zn_2_GeO_4_‐based NTE materials [96]. These collective motions induce correlated disorder around the interstitial oxygen species and modulate the accessible migration space, thereby coupling low/negative thermal expansion with interstitial oxide‐ion migration in Zn_2‐_ *_x_* La*_x_* GeO_4+_ *_x_*_/2_.

### 3.4 Oxide‐Ion Migration Dynamics

To study the oxide ion migration mechanisms in Zn_2‐_ *_x_* La*_x_* GeO_4+_ *_x_*_/2_, we performed bond‐valence site energy (BVSE) calculations based on both the undoped parent Zn_2_GeO_4_ and the refined NPD structure of Zn_1.96_La_0.04_GeO_4.02_. In the parent Zn_2_GeO_4_ structure, the calculated migration barriers (Figure S13) are relatively high: 0.712 eV for two‐dimensional (2D) and 1.305 eV for three‐dimensional (3D) migration, consistent with the limited intrinsic oxide‐ion mobility inferred experimentally (Figure S4). In contrast, the La‐doped Zn_1.96_La_0.04_GeO_4.02_ shows a substantially reduced 2D and 3D migration barrier of 0.428 eV (Figure 6a), highlighting the role of interstitial oxygen introduced by La doping in facilitating long‐range ion transport. BVSE maps viewed along the [001] direction (Figure 6b) reveals continuous, low‐energy oxide‐ion migration pathways extending across the framework. In the *ab* plane (Figure 6b), long‐range diffusion occurs through interconnected 4‐ and 6‐membered rings composed of ZnO_4_ and GeO_4_ tetrahedra, forming a two‐dimensional conduction network. Additionally, pathways in the *bc* plane (Figure 6c) indicate further connectivity through shared oxygen vertices, suggesting a three‐dimensional percolation of oxide‐ion transport across the lattice.

**FIGURE 6:**
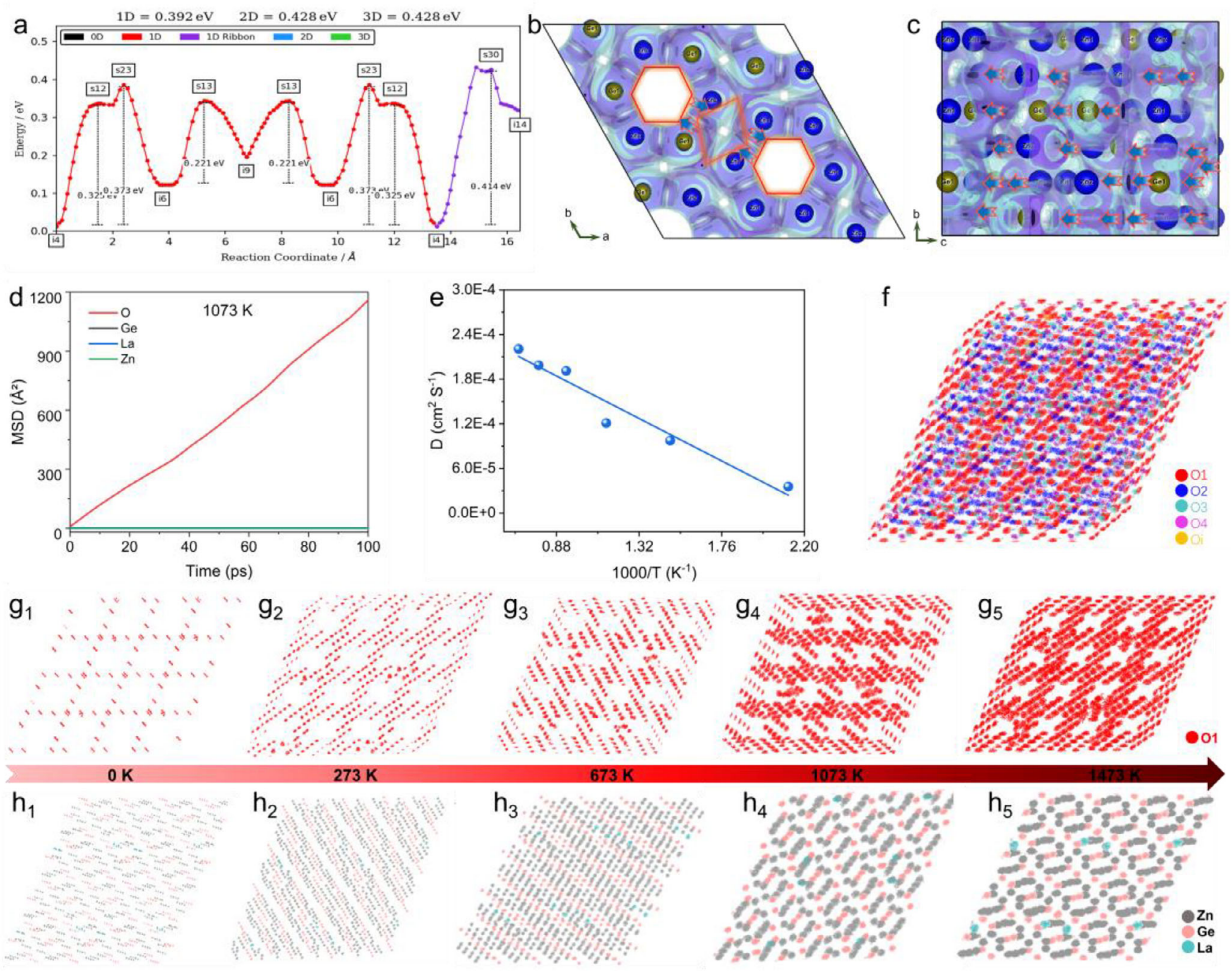
Oxide‐ion migration dynamics in Zn_2‐_ *_x_* La*_x_* GeO_4+_ *_x_*_/2_. (a) BVSE‐modeled migration barriers in one‑, two‑, and three‑dimensional pathways. (b,c) BVSE maps of oxide ions with an iso‐surface of 0.428 eV, viewed along the (b) [001] and (c) [100] directions. Arrows denote the 2D and 3D migration pathways within the open‐ring framework. (d) MSD values of Zn, La, Ge, and O atoms as a function of simulation time at 1073 K. (e) Arrhenius plot of oxide‐ion diffusion coefficients. (f) Scatter plot of oxygen atoms including O1, O2, O3, O4, and interstitial O_i_. (g_1‐5_, h_1‐5_) Temperature‐dependent scatter plots of (g_1‐5_) O1 atoms and (h_1‐5_) cation atoms (Zn, La, Ge) from 0 to 1473 K.

To comprehensively account for the polyhedral interactions and the impact of defect concentrations on oxide ion transport, we performed molecular dynamics simulations based on the interatomic potential method to further explore the oxide ion transport mechanism in Zn_2‐_ *_x_* La*_x_* GeO_4+_ *_x_*_/2_. The employed potential energy parameters (Table 1) accurately reproduced the experimental structural parameters of Zn_2_GeO_4_ (Table S2), validating their suitability for modeling this system. Mean square displacement (MSD) values of both cations and oxide ions in Zn_2‐_ *_x_* La*_x_* GeO_4+_ *_x_*_/2_ (Figure 6d) reveal that oxide ions show significant long‐range migration, whereas the cations are confined to lattice vibrations. The calculated diffusion coefficients (Figure 6e) range from 3.56×10^−5^ cm^2^ S^−1^ to 2.21×10^−4^ cm^2^ S^−1^ over the 273–1473 K range, indicating the favorable oxide ion mobility within the NTE Zn_2‐_ *_x_* La*_x_* GeO_4+_ *_x_*_/2_ framework.

Scatter plots of atomic positions for Zn_2‐_ *_x_* La*_x_* GeO_4+_ *_x_*_/2_ (Figure 6f) provide further evidence that oxygen positions are involved in oxygen exchange, whereas cation positions without any cationic exchange, consistent with the MSD results (Figure 6d). Temperature‐dependent scatter plots of the O1 sublattice (Figure 6g_1_‐g_5_) and cation sublattice (Figure 6h_1_‐h_5_) over the temperature range of 0–1473 K reveal that oxide ions migrate from 4‐membered rings to 6‐membered rings (Figure 6g_1_‐g_5_), while no detectable cation migration is observed (Figure 6h_1‐5_). Similar ring‐to‐ring migration behavior is also observed for other oxygen sublattices (Figures S14 and S15). Radial distribution functions (RDFs) for Zn─O and Ge─O interactions (Figures S16–S18), derived from MD simulations, reveal dynamic reconfiguration of local coordination in Zn_2‐_ *_x_* La*_x_* GeO_4+_ *_x_*_/2_. For the Zn2‐O interactions over the 0–100 *ps* (Figure S16), the initially coordinated oxygen atoms (O1, O3, O4) surrounding Zn2 are progressively supplemented by oxygen atoms at additional sites (O2, O_i_), evidencing dynamic oxygen coordination exchange. Analogous behavior is observed for Zn1‐O (Figure S17) and Ge─O (Figure S18) interactions, highlighting the participation of multiple oxygen species in the evolving local coordination environment that facilitates the long‐range oxide ion migration.

## 4 Conclusions

In summary, we provide a phonon‐driven NTE flexible framework design paradigm that harnesses the intrinsic lattice flexibility to concurrently suppress thermal expansion and activate oxide‐ion conduction over a broad temperature window. We demonstrated its feasibility through the development of interstitial oxide‐ion conducting NTE solid electrolytes Zn_2‐_ *_x_* La*_x_* GeO_4+_ *_x_*_/2_, with interstitial oxide ions as the dominant mobile species. The interstitial oxygen was accommodated within the 4‐membered rings rather than the 6‐membered rings, with its correlated disorder of interstitial oxygen predominantly coordinated to the GeO_4_ environment. These phonon‐driven NTE behaviors that transverse thermal vibrations and collective distortions of the surrounding flexible GeO_4_ and ZnO_4_ tetrahedra simultaneously modulate the migration dynamics of interstitial oxygen defects, enabling long‐range oxide‐ion migration from 4‐membered to 6‐membered rings. Notably, Zn_1.96_La_0.04_GeO_4.02_ combines interstitial oxide‐ion transport with ultralow bulk thermal expansion coefficients (α_V_ = 7.232 × 10^−6^ K^−1^, α_a_ = α_b_ = 1.823 × 10^−6^ K^−1^, α_c_ = 3.570 × 10^−6^ K^−1^) and near‐zero macroscopic linear thermal expansion (α_L_ = 0.307 × 10^−6^ K^−1^) over a broad temperature window. To the best of our knowledge, this α_V_ value is the lowest reported bulk thermal‐expansion coefficient among oxide‐ion‐conducting solids. More broadly, this work identifies a previously unexplored design route to couple phonon‐driven NTE frameworks with ion‐transport functionality, providing a promising strategy for TEC management in next‐generation solid‐state ionic devices.

## Author Contributions

X. Kuang, X. Xing, and X. Li conceived and supervised the project, and prepared and revised the manuscript. X. Li carried out the syntheses and characterization of samples, as well as data analysis. L. Liang and Q. Shi performed and analyzed the experimental measurements. X. Wang performed the RED data collection. C. Li helped collect the neutron pair distribution function. S. Deng and L. He helped collect the neutron diffraction data. K. Lin and Q. Li provided thoughtful and constructive comments on this manuscript. All authors reviewed and approved the final version of the manuscript.

## Conflicts of Interest

The authors declare no conflicts of interest.

## Supporting information




**Supporting File**: advs76792‐sup‐0001‐SuppMat.docx.

## Data Availability

The data that support the findings of this study are available from the corresponding author upon reasonable request.
