# Supplementary material for: Phonon‐Driven Tetrahedral Tilts Enable Ultralow Bulk Thermal Expansion and Interstitial Oxide‐Ion Migration in Phenacite Solid Electrolytes
Source: Adv Sci (Weinh). 2026 Jul 27;13(58):e76792. doi: 10.1002/advs.76792 (PMC13403737; doi:10.1002/advs.76792)
Supplement: Supplementary file 1 [file ADVS-13-e76792-s001.docx]

**Supplementary Information**

**Phonon-Driven** **Tetrahedral Tilts Enable Ultralow Bulk Thermal Expansion and Interstitial Oxide-Ion Migration in** **Phenacite Solid Electrolytes**

Xiaohui Li,^a^* Lu liang,^b^ Qilong Shi,^a^ Xiaoge Wang,^c^ Cheng Li,^d^ Sihao Deng,^e^ Lunhua He,^e,f^ Qiang Li,^b^ Kun Lin,^b^ Xianran Xing,^b^* and Xiaojun Kuang^a,g^*

^a^ Guangxi Key Laboratory of Electrochemical and Magnetochemical Functional Materials, College of Chemistry and Bioengineering, Guilin University of Technology, Guilin 541004, People’s Republic of China.

^b^ Beijing Advanced Innovation Center for Materials Genome Engineering, Institute of Solid State Chemistry, University of Science and Technology Beijing, Beijing 100083, People’s Republic of China.

^c^ College of Chemistry and Molecular Engineering, Peking University, Beijing National Laboratory for Molecular Science, Beijing 100871, People’s Republic of China.

^d^ Oak Ridge National Laboratory, Neutron Sciences Directorate, Oak Ridge, TN, 37831-2008, United States of America.

^e^ Songshan Lake Materials Laboratory, Dongguan 523808, China; Spallation Neutron Source Science Center, Dongguan 523803, People’s Republic of China.

^f^ Beijing National Laboratory for Condensed Matter Physics, Institute of Physics, Chinese Academic of Sciences, Beijing 100190, People’s Republic of China.

^g^ Future Energy Interdisciplinary Center, Key Laboratory of Solid-State Energy Conversion and Storage of Jiangxi Education Department, School of Intelligent Manufacturing and Future Energy, Gannan Normal University, Ganzhou, Jiangxi 341000, People’s Republic of China.

E-mail: [xiaohuili@glut.edu.cn](mailto:xiaohuili@glut.edu.cn); xing@ustb.edu.cn; kuangxiaojun@gnnu.edu.cn

**Figure S1.** Rietveld refinement plots of XRD pattern of Zn_1.96_La_0.05_GeO_4.025_.

**Table S1.** The refined structural parameters of Zn_1.96_La_0.04_GeO_4.02_ from XRD data*.

| Atom | Wyckoff site | *x* | *y* | *z* | Occ. | B_iso_(Å^2^) |
| --- | --- | --- | --- | --- | --- | --- |
| Zn1 | 18f | 0.78574(56) | 0.97428(63) | 0.41355(71) | 0.94 (3) | 1.4(5) |
| La1 | 18f | 0.78574(56) | 0.97428(63) | 0.41355(71) | 0.06 (3) | 1.4(5) |
| Zn2 | 18f | 0.78815(55) | 0.97820(42) | 0.07980(53) | 1 | 1.6(4) |
| Ge1 | 18f | 0.78568(48) | 0.98025(36) | 0.74513(61) | 1 | 1.2(4) |
| O1 | 18f | 0.7989(18) | 0.9183(17) | 0.9152(35) | 1 | 1 |
| O2 | 18f | 0.7958(21) | 0.9167(18) | 0.6070(18) | 1 | 1 |
| O3 | 18f | 0.77923(27) | 0.8883(13) | 0.2584(33) | 1 | 1 |
| O4 | 18f | 0.6676(18) | 0.9932(15) | 0.7263(26) | 1 | 1 |

* Space group: *R*$\bar{3}$, *a* = *b* = 14.2368(1) Å, *c* = 9.5266(1) Å, *α* = *β* = 90º, *γ* = 120º, *V* = 1672.23(3) Å^3^.

**Figure S2.** EDS spectra of Zn_1.96_La_0.04_GeO_4.02_.

**Figure S3.** TEM elemental mapping and EDS spectra of Zn_1.96_La_0.04_GeO_4.02_.

**Figure S4.** The complex impedance plots of parent Zn_2_GeO_4_ at 750 ºC.

**Figure S5.** Complex impedance plots of Zn_1.96_La_0.04_GeO_4.02_ at 550 ºC

**Supplementary Note 1.** Rationale and physical interpretation of the equivalent-circuit fitting.

The equivalent-circuit model was selected according to the characteristic features of the Nyquist plots and the typical impedance response of polycrystalline ceramic electrolytes. Since real ceramic electrolytes often show non-ideal relaxation behavior due to microstructural heterogeneity, grain-boundary effects, and distributed relaxation times, depressed semicircles are commonly observed instead of ideal RC arcs. Therefore, constant-phase elements (CPEs) were used to describe the non-Debye-type capacitive responses, following established treatments of impedance relaxation and universal dielectric response.[1] In the present model, the high-frequency and intermediate-frequency components are assigned to the bulk and grain-boundary responses of the electrolyte, respectively. This assignment is supported by their characteristic capacitance values, which are within the typical ranges for intrinsic lattice and grain-boundary processes. The low-frequency Warburg-type element is attributed to the electrode/electrolyte interfacial process, where ion accumulation and interfacial diffusion dominate under ion-blocking electrode conditions.[2] Thus, the selected equivalent circuit provides a reasonable separation of bulk, grain-boundary, and electrode/electrolyte interfacial contributions. Similar equivalent-circuit treatments have been widely used for ceramic ionic conductors and related electroceramic materials.[3-5]

**Figure S6.** The complex impedance plots of Zn_1.96_La_0.04_GeO_4.02_ at 600 ºC under different oxygen partial pressures (pure O_2_, Air, and 1%O_2_).

**Figure S7.** Variable-temperature XRD patterns of Zn_1.96_La_0.04_GeO_4.02_ in the temperature range of 25-1000 ºC.

**Figure S8.** TG-DSC curves of Zn_1.96_La_0.04_GeO_4.02_.

**Figure S9.** Cell parameters as a function of temperature.

**Figure S10.** Linear thermal expansion of Zn_1.96_La_0.04_GeO_4.02­_ ceramic.

**Figure S11.** Coordination-number distributions of Zn, La, and Ge with respect to neighboring oxygen atoms in the RMC-refined big-box model.

**Figure S12.** Calculated (a) phonon dispersion and (b) projected phonon density of states of Zn_2_GeO_4_ framework.

**Figure S13.** BVSE-modeled migration barriers for the parent Zn_2_GeO_4_­ along one‑, two‑, and three‑dimensional pathways.

**Table S2.** Calculated and experimental structural parameters for Zn_2_GeO_4_.

| Parameters | Calculated | experimental | ∆（Calc.-Exp.） |
| --- | --- | --- | --- |
| *a*(Å) | 14.113798 | 14.2775 | -0.163702 |
| *b*(Å) | 14.113798 | 14.2775 | -0.163702 |
| *c*(Å) | 9.667797 | 9.55481 | 0.112987 |
| α(°) | 90 | 90 | 0 |
| β(°) | 90 | 90 | 0 |
| γ(°) | 120 | 120 | 0 |
| Volume(Å^3^) | 1667.807641 | 1686.774846 | -18.967205 |
| Zn1-O2 | 2.00473 | 1.9873 | 0.01743 |
| Zn1-O2 | 1.9939 | 1.9799 | 0.014 |
| Zn1-O3 | 1.95257 | 1.91124 | 0.04133 |
| Zn1-O4 | 1.95673 | 1.94546 | 0.01127 |
| Zn2-O1 | 2.00625 | 1.9885 | 0.01775 |
| Zn2-O1 | 1.98254 | 1.92302 | 0.05952 |
| Zn2-O3 | 1.94668 | 1.9936 | -0.04692 |
| Zn2-O4 | 1.92208 | 1.94594 | -0.02386 |
| Ge1-O1 | 1.7342 | 1.7619 | -0.0277 |
| Ge1-O2 | 1.7383 | 1.7597 | -0.0214 |
| Ge1-O3 | 1.75202 | 1.7413 | 0.01072 |
| Ge1-O4 | 1.72936 | 1.7676 | -0.03824 |

**Figure S14.** Scatter plot of oxide ions positions including O2, O3, O4, and Oi in the temperature range of 0-670 K.

**Figure S15.** Scatter plot of oxide ions positions including O2, O3, O4, and Oi in the temeprature range of 873-1473 K.

**Figure S16.** RDFs of Zn2-O interactions with the simulation time.

**Figure S17.** RDFs of Zn1-O interactions with the simulation time.

**Figure S18.** RDFs of Ge1-O interactions with the simulation time.

**Reference**

[1]	J. S. Macdonald, Note on the parameterization of the constant-phase admittance element, *Solid State Ionics* **1984**, *13*, 147.

[2]	J. T. S. Irvine, D. C. Sinclair, A. R. West, Electroceramics : Characterization by Impedance Spectroscopy, *Adv. Mater.* **1990**, *2*, 132.

[3]	N. Masó, A. R. West, Electronic Conductivity in Yttria-Stabilized Zirconia under a Small dc Bias, *Chem. Mater.* **2015**, *27* (5), 1552.

[4]	N. Masó, X. Y. Yue, T. Goto, A. R. West, Frequency-dependent electrical properties of ferroelectric BaTi_2_O_5_ single crystal, *J. Appl. Phys.* **2011**, *109* (2), 024107

[5]	E. J. Abram, D. C. Sinclair, A. R. West, A Strategy for Analysis and Modelling of Impedance Spectroscopy Data of Electroceramics: Doped Lanthanum Gallate, *J. Electroceram.* **2003**, *10*, 165.
